# Dietary consumption and its effect on nutrition outcome among under-five children in rural Malawi

**DOI:** 10.1371/journal.pone.0237139

**Published:** 2020-09-14

**Authors:** Kennedy Machira, Tobias Chirwa

**Affiliations:** 1 Lilongwe University of Agriculture and Natural Resources, Faculty of Development Studies, Lilongwe, Malawi; 2 Witwatersrand University, School of Public Health, Education Campus, Park Town, Johannesburg; Institute of Economic Growth, INDIA

## Abstract

**Background:**

Despite remarkable progress in reducing under five mortalities in Malawi, a relative proportion of under nutrition among children still exist.

**Objectives:**

The study examines dietary consumption factors and their effect on under nutrition outcomes among children under five years’ children in rural Malawi.

**Methods:**

Using the 2015–16 Malawi Demographic and Health Survey, in which 4,150 children were reported of under nutrition statuses, the study used nested logistic regression models to estimate factors influencing the prevalence of under nutrition among children.

**Results:**

Based on the results, exclusive breast feeding among children under five years, after controlling for parental socioeconomic factors, was found to reduce the levels of wasting (ODDS RATIO [OR] = 0.763; p < 0.05), underweight (OR = 0.548; p < 0.001) and stunting (OR = 0.709; p < 0.005). Furthermore, it was found that despite the perceived adequacy among women in accessing fruits and vegetables, carbohydrates and micro-nutrient supplements, their children under five years, still experiences public health challenges and suffers from wasting, underweight and stunting.

**Conclusion:**

There is need to implement extensive pro-rural under five nutritional and health educational advocacy using community-based approaches, targeting parents, emphasizing the significance of exclusive breast feeding and consistencies in giving dietary foods, if and only if the persistent public health challenges due to under nutrition among children under five years, is to be sustainably dealt with, in Malawi.

## Background

In most developing countries, Malawi included, under nutrition remains the public health challenge, which has a dare consequence on the children in the first five years of life [1]. For instance, it is argued that the state of under nutrition, still, attributes to nearly half of all under-five deaths. A recent nutritional report posits that, in 2016 alone, about 155 million of children under-five are stunted. On the same note, about 52 million of them still experience wasting [2]. Worth to note that challenges to access of poor nutritive foods and micronutrients among most children in the first 1000 days, not only result in creating an irreversible bodily impaired cognitive ability among them, but also affected the construct of their performance capacity [1], factors which affect their well-being development. It has been postulated that under nutrition, more especially among the children under five, is attributed by an intertwined of diverse social factors, such as low parental knowledge on child care on best feasible feeding habits, inadequacy understanding of suitable feed mix for children, inconsistent timing of exclusive breast feeding in addition to low interface of women to health care facilities after delivery to engage on under nutrition for improved maternal and child nutritional statuses [3–6]. These factors, if not checked, reduces quality of life among children [1].

In view of the perceived under nutrition child health challenges, the congress of the World Health Organisation in 2013, advocated for renewed efforts among developing countries, more especially experiencing public health under nutrition challenges, to scale up on country’s national nutrition policies in order to improve the long standing under five under nutrition burden [7]. Such under nutrition policy advocates should include strengthening intra-nation public health strategies aimed at reducing developing countries under nutrition status by 2025.

However, in Malawi, despite adoption of the public health policies, among them the National Nutrition Policy, which aimed at championing a quest to improving under nutrition challenge affecting the children in order to acquire long term gain in reducing morbidity, mortality and associated disability [7,8], the country’s under nutrition fight is far from being won. This is despite, the country’s remarkable progress gained in reducing under-five mortality [9–13]. For instance, about 48% of the under-five children in Malawi do suffer from under-nutrition’s [14]. Additionally, the government implemented a multi-sectoral nutrition initiative, among one, the promotion of mothers’ exclusive breast feeding, an Integrated Management of Childhood Illness (2005–06) [15]; infant and youth Child Nutrition Policy (2005) [16], National Plan of Action for Nutrition (2000) [17], Malawi Growth and Development Strategy Policy I (2006) and II (2017) [18,19], to enhance child health nutritional outcomes. Despite such policies and initiatives, proportion of the children still suffers from wasting (3.8%), stunting alarmingly at 42.4% and underweight (16.7%) weighted from 2.8 million registered under-five children population, nationally (NSO and ICF Macro, 2015–16). Also, to note, about 87% of these children’s parents’ households, are predominantly resident in rural areas [19].

Therefore, the paper examined the effect of dietary and socioeconomic factors on the prolonged undernutrition among children under five in rural Malawi. This is amidst the country’s contrary continuum gains in reducing children under five mortalities, nationally.

## Methods

### Data sources

The Study used Demographic and Health Survey Data 2015–16, a nationally representative data that collect data different categories of data that include nutrition and health of the women of reproductive ages between 15 and 49 years. Before the authors accessed the data, an electronic form was filled which requested them to enter the proposal of this work. The ethics committee of DHS authenticated the study and give them the privilege to download the data on their website after accepting terms and conditions on data sharing and use policy. The ethical approval for DHS data was collected by ICF International Institutional Review Board (IRB) and Malawi's National Statistics Office, as they have work relationship on data collection in the country. These two bodies allow scholars to use their collected waves of data for further analysis, which in this case, the team is falling in. During the data collection, respondents are provided with a verbal informed consent prior to each interview and are identifiable within the data set. Therefore, for the current study, further approvals were not required as use of the data is authorized by ICF and NSO Malawi which are data custodians and that data is open for public use.

### Sampling frame and sampling criteria

The 2015–16 MDHS data adopted the sampling frame that the country used to collect 2008 Malawi National Population and housing Census. In 2008 census, a total of 850 standard enumeration areas (SEA) of which 173 SEA were urban based and 677 were the rural counterpart. The study used both stratified and cluster sampling techniques to sample women who participated in the study. About 21 370 rural households were interviewed in which 19, 315 eligible women were interviewed between ages 15–49, upon consent information about their children health statuses were collected. As such, a total of 4151 children had their nutrition status reported by their mothers.

### Selection criteria

The study considered used individual women data from ICF and Macro between the ages 15–49. Upon visiting the health care facilities, about 4151 children were reported to have been checked of the anthropometric attributes such as undernutrition, stunting and wasting. Therefore, any children whose mother never visited the health care facilities and have their anthropometric measure did not form part of the study sample.

### The study

Using Anthropometric measure collected indicators in which weight-for-age, height-for-age and weight- for-height were populated. The study adopted the WHO 2006 standard definition which highlighted that z-score for weight-for-age (WAZ) below -2 standard deviation (SD) is defined as the child under-nutritional status as underweight whereas z score for height-for-age (HAZ) below -2SD as stunting and for weight-for-height (WHZ) -2SD as wasting. Out of this variables, under-nutrition measures were defined as a binary variable in which if the Z score of WAZ, HAZ and WHZ was less than -2SD, was recoded as a 1 to denote under-nutrition, namely, underweight, stunting and wasting respectively.

Other independent variables were adopted from the MDHS to derive the dietary consumption and the socio-economic variables of the parents. As most of the dietary consumption indicators were many, a principal component analysis (PCA) technique was used to create factor variables that were defined as *animal protein consumption*, if a child was reported to be given any food from comprising of the animal proteins such as eggs, meat, fish; *fruit and vegetables*, if a child was reported being given fruits such as mango, pawpaw, and vegetables such as Chinese vegetables, rape, cabbage among others; and *carbohydrates consumption*, if a child was reported to have been feed on porridge, bread, rice, pap, pasta among others. On the same note other variables such as micronutrients supplement and modern fortified baby food such as Ceralac, among others were recoded 1 if a child was fed with each of these and 0 for otherwise. Furthermore, exclusive breast feeding status was also taken and was measured as 1 if the child was exclusively breast feed at any point after birth and 0 for otherwise. In term of the socio-economic status of the parents, maternal and paternal education was adopted and measured based on level attained as follows: no education, primary level education and secondary education and above. On the same note, behavioural attributes of the mothers to seek child health care was measured as a proxy of using postnatal care services after child delivery and was recoded as 1 if postnatal care services were patronised and 0 for otherwise. Finally, the woman was requested to indicate the parity of the child and this was defined as 1 if the child parity was 1 and 2 and higher for otherwise.

## Analysis

The study used descriptive analysis in which the frequencies and percentages were used to explain the attributes given by the respondents. On the same way, a chi-square test of association was performed to measure the significance between independent and dependent variables. Further to this, two nested multivariate logistic regressions were modelled to estimate factors affecting under-nutritional status among children the under five in rural Malawi. This was suggested in order to test if there is consistent effect of the covariates affecting under nutrition among children as the other control variables are included in model which is not a common practice in the traditional multivariate logistic regression. For instance, in the first nested model, child’s dietary consumptions factors were regressed to estimate its effect on under-nutrition condition among the under-five. In the second nested model, the same dietary consumption factors controlling for parental socio-economic factors in order to test variability effect of the dietary consumption factors while testing its effect on under-five under nutritional status. In trying to understand the determinants affecting the undernutrition outcome, children under five years were further decomposed by ages in months as 0–5, 6–23 and 26–59. This decomposition approach has an impetus to understand more pronounced determinants affecting outcome relative to aggregated counterpart [20–22].

The analysis for the study was conducted using STATA 15.0 [23].

## Results

### Background attributes of the sample attributes

As indicated in Table 1, the results show that summary information found that in terms of the animal protein consumption, a relatively equal proportion of the children under nutrition have inadequate animal protein consumption with a mean of 83% across. Similarly, on fruits and vegetables a mean of 69% of the women that their underweight children were inadequately consuming the fruits and vegetables in their meals. This inadequacy was also noted on the carbohydrate related food consumption.

**Table 1:** Background characteristics by nutritional status of rural children under-five.

	Wasting		Under-weight		Stunting	
	N = 3469	% = 100	N = 3467	% = 100	N = 3460	% = 100
***Dietary Consumption Factors***						
**Animal Protein**						
Inadequate	2887	83.2	2885	83.2	2878	83.2
Adequate	582	16.8	582	16.8	582	16.8
**Fruit and vegetables**						
Inadequate	2408	69.4	2406	69.4	2399	69.3
Adequate	1061	30.6	1061	30.6	1061	30.7
**Carbohydrate consumed**						
Inadequate	2532	73.0	2529	72.9	2523	72.9
Adequate	937	27.0	938	27.1	937	27.1
**Exclusively breastfed the child**						
No	1772	51.1	1769	51.0	459	13.3
Yes	1697	48.9	1698	49.0	3001	86.7
**Micronutrient supplement**						
Inadequate	3414	98.4	3412	98.4	3405	98.4
Adequate	55	1.6	55	1.6	55	1.6
**Fortified baby fed (Ceralac)**						
Inadequate	3414	98.4	3412	98.4	3360	97.1
Adequate	55	1.6	55	1.6	100	2.9
***Parental factors***						
**Maternal education**						
No education	482	13.9	476	13.7	476	13.8
Primary	2380	68.6	2383	68.7	2377	68.7
Secondary and higher	607	17.5	608	17.5	607	17.5
**Paternal education**						
No education	885	25.5	879	25.4	879	25.4
primary	1699	49.0	1701	49.1	1696	49.0
Secondary and higher	885	25.5	887	25.6	885	25.6
**Postnatal care service visit**						
No	1996	57.5	1994	57.5	1990	57.5
Yes	1473	42.5	1473	42.5	1470	42.5
**Parity of child**						
1	767	22.1	769	22.2	768	22.2
> = 2	2702	77.9	2698	77.8	2692	77.8

In as far as micronutrients consumption and fortified baby feeding are concerned, the study it is observed almost a negligible proportion of the children about 2% of children under five were adequate given these supplement and foods by their mothers. This is partly due to inadequate resources among the rural women to adequately access supplementary foods for their children which oftentimes regarded as luxurious [24]. Considering the socio-economic attributes of the parents, it was found that about 68.7% of the children were from women that had attained up to a primary school education level. It is also worth noting that less than 20% of the children in rural Malawi were from parents who attained secondary education and above. In terms of the postnatal care services, about 57.5% of the women had no health seeking behaviour after child birth and about 78% of them had parity greater and equal to 2. Table 1 provides more detail.

### Dietary consumption and parental socio-economic factors in relation with under nutrition of children under-five in rural Malawi

Table 2 presents the Pearson measure of association between dietary and socioeconomic independent factors with the under-nutrition dependent variables. Based on the results, the study found that the rural children under nutrition related to different dietary factors. For instance, inadequate consumption of animal protein consistently and significantly relates to wasting (*χ*^2^ = 4.1,*p*<0.05), underweight (*χ*^2^ = 5.1,*p*<0.05) and stunting *χ*^2^ = 11.9,*p*<0.05). On the same note, inadequate consumption of fruits and vegetables among the rural under-five children related significantly with wasting (*χ*^2^ = 6.5,*p*<0.05), underweight (*χ*^2^ = 4.5,*p*<0.05) and highly on stunting (*χ*^2^ = 11.5, *p*<0.001). However, in terms of carbohydrates consumption inadequacy, it related significantly on wasting (*χ*^2^ = 5.3,*p*<0.05) and stunting alone whereas exclusive breast feeding practices adequacy significantly related to underweight and stunting under-nutritional conditions. Considering the use of fortified baby food, inadequate consumption of the fortified foods had had a significant association with stunting the under-five children. On the parental characteristics, it was found that maternal education related significantly on underweight (*χ*^2^ = 12.4,*p*<0.05) and stunting *χ*^2^ = 10.4,*p*<0.05). On the same note, paternal education status had had a significant association with underweight situation of the children under-five in rural Malawi (*χ*^2^ = 12.4,*p*<0.05). Table 2 presents more details.

**Table 2:** Pearson’s Chi-square results of the measure of association between explanatory variables and nutrition outcome, namely, wasting, underweight and stunting among rural children.

Variables	Wasting	Underweight	Stunting
***Dietary consumption Factors***			
Animal Protein	4.1**	5.1**	11.9**
Fruit and vegetables	6.5**	4.5**	11.5***
Carbohydrate consumed	5.3**	2.6	3.9**
Exclusively breastfed the child	1.4	69.9***	49.5***
Micronutrient supplement	0.2	1.4	0.8
Fortified baby fed (Ceralac)	0.2	1.4	2.9*
*Parental factors*			
Maternal education	0.16	12.4**	10.4**
Paternal education	3.7	4.7*	2.5
Postnatal care service visit	0.1	2.5	1.2
Parity of child	0.09	2.1	0.0

*** p < 0.001

** p < 0.05 and

*p < 0.1.

### Multivariate analysis

In the multivariate approaches, namely, within and between in order to establish which variables consistently affects under-nutrition among under-five children in rural Malawi. Based on the nested logistic regression in which dietary consumption factors were modelled to estimate their influence on under-nutritional status of rural children under-five in Malawi in the first iteration. In the second iteration, the prior dietary consumption factors were re-modelled while controlling for parental socio-economic attributes. Based on the two models, the study found that variations still exist on both dietary consumption and socio-economic factors influence of under-nutrition status among the under-five in rural Malawi.

As is illustrated in Table 3, unadjusted models indicate that inadequate consumption of fruits and vegetables was the more likely factor that not only increased wasting under-nutritional status of children under five at 10% levels (OR = 1.242, p < 0.1), but also increased underweight conditions moderately (OR = 1.532, p < 0.05) compared to their counterparts that that were adequately fed with fruits and vegetables. In terms of carbohydrates consumption, it was found inadequate consumption of carbohydrates predicted both increased likelihood of both underweight (OR = 1.421, p < 0.05) and stunting (OR = 1.623, p < 0.001) under-nutritional status among children under-five in rural Malawi. With regard to exclusive breast feeding, unadjusted model indicates that intensifying breast feeding in rural Malawi, had a significant decreasing effect on the levels of wasting among children under-five in rural Malawi (OR = 0.693, p < 0.001). Despite this remarkable improvement in reducing wasting condition among the under-five, in subsequent unadjusted logistic models, exclusive breast feeding had had little effect to significantly reduce under-weight child status (OR = 1.697, p < 0.001) and stunting (OR = 3.216, p < 0.05). Similarly, inadequate consumption of micro-nutrients supplements among the under-five increased the likelihood of under-nutrition condition in form of under-weight (OR = 3.38, p<0.05) and stunting (OR = 5.489, p < 0.001).

**Table 3:** Nested logistic regression estimating factors influencing nutritional status of rural children under-five in Malawi.

Variables	Nutrition status of the children under-five					
	Wasting		Under-weight		Stunting	
	Model 1	Model 2	Model 1	Model 2	Model 1	Model 2
	OR	OR	OR	OR	OR	OR
***Dietary Consumption Factors***						
**Animal Protein**						
Inadequate	1	1	1	1	1	1
Adequate	1.108	1.087	0.95	0.924	0.862	0.749*
**Fruit and vegetables**						
Inadequate	1	1	1	1	1	1
Adequate	1.242*	1.29**	1.532**	1.243	1.512	1.032
**Carbohydrate consumed**						
Inadequate	1	1	1	1	1	1
Adequate	1.155	1.145	1.421**	1.217	1.623***	1.138
**Exclusively breastfed the child**						
No	1	1	1	1	1	1
Yes	0.693***	0.763**	1.697***	0.548***	3.216***	0.709**
**Micronutrient supplement**						
Inadequate	1	1	1	1	1	1
Adequate	1.025	1.133	3.338**	1.8	5.489***	2.234
**Fortified baby fed (Ceralac)**						
Inadequate	1	1	1	1		1
Adequate	0.895	0.954	0.907	0.901		1.031
***Parental factors***						
**Maternal education**						
No education		1		1		1
Primary		1.035		1.847***		2.418***
Secondary and higher		1.105		1.1667***		1.515
**Paternal education**						
No education		1		1		1
Primary		0.832*		1.879***		2.204
Secondary and higher		0.739**		1.682***		2.015
**Postnatal care service visit**						
No		1		1		1
Yes		0.961		1.349**		1.415**
**Parity of child**						
1		1		1		1
> = 2		1.013		1.371***		1.91***

*** p < 0.001

** p < 0.05 and

*p < 0.1.

Model 1: unadjusted model.

Model 2: adjusted model after controlling for parental factors.

In adjusted models after controlling for parents’ socio-economic status, the study found that exclusive breast feeding behavioural practices remained a significant factor that reduced wasting (OR = 0.763, p < 0.05), underweight (OR = 0.548, p < 0.001) and stunting (OR = 0.709, p < 0.05). In as far as education is concerned; it was found that primary education attainment by women is not helping in providing better understanding among them to improve quality of health life of their under-five children in rural Malawi. The current study found out that despite women in rural Malawi do have opportunities to attain primary school education, the degree of children under five years experiencing an under nutrition condition remain high. For instance, the study found that both underweight and stunting condition among the children was higher and affected greatly on their health statuses and significantly noted with an OR = 1.847, p < 0.001 and OR = 2.418, p < 0.001, respectively. On the same note, there is a slight variation the influence of paternal education attainment on the under-nutrition status of the children under-five. The study revealed that paternal education has a significant effect in reducing wasting condition among the under-five. Yet, it is contrary in having a similar impact among those with underweight nutritional health status. In as far as health seeking behaviour its effect on under nutritional condition is concerned, the study found that despite women having an opportunity to seek postnatal care services after childbirth, its relative impact in no positive effect to reduce under-weight (OR = 1.349, p < 0.05) and stunting (OR = 1.415, p < 0.05). In terms of the parity, the women that had parity of 2 children and higher were associated with significant and increased odds of having their children experience underweight nutritional condition (OR = 1.371, p < 0.001) and stunted (OR = 1.91, p < 0.001) relative to their counterpart with a parity of 1 in both cases.

### Decomposing determinants affecting nutrition outcome among the children under-five by age category 0–5 months, 6–23 months and 26–59 months

Therefore, considering the ages of the children under-five categorised between 0–5, 6–23 and 26–59 months, the study found exclusive breast feeding as the predominant behavioural determinants affecting nutrition outcome among the children under-five by ages. For instance, exclusive breast feeding was found to reduce problems of under-weight among children aged 0–5 months old (OR = 0.63, p<0.001) contrary to the children ages between 6–23 months old due to little effect in reducing significantly under-weight condition among the children (OR = 3.89, p<0.001). In terms of the parents feeding support on their children with fortified food supplement, it was found the consumption of the fortified food among the children had little effect as the fortified food dietary increased the likelihood among the children’s underweight public health challenge for ages 6–23 months (OR = 2.01, p <0.001) and 24–59 months (OR = 6.21, p<0.001). In terms of the stunting, the study found that exclusive breast feeding between children aged 0–5 month (OR = 2.68, p < 0.001) and 6–23 months old (OR = 3.89, p <0.001) increased the likelihood of stunting among the children significantly. Similarly, considering the fortified food supplements the study found that it had increased the likelihood on children aged 6–23 months (OR = 1.67, p< 0.001) and 24–59 months old (OR = 10.82, p<0.001). However, the study found that exclusive breast feeding as fundamental behavioural predictor that reduced significantly wasting amongst children aged between 0 and 5 months old (OR = 0.82, p<0.001). Table 4 illustrates more detail.

**Table 4:** Dietary factors affecting nutrition outcome by rural children ages ranging from 0–5, 6–23 and 24–59 months.

variables	Underweight			Stunting			Wasting		
**Dietary factors**	**0-5m**	**6-23m**	**24-59m**	**0-5m**	**6-23m**	**24-59m**	**0-5m**	**6-23m**	**24-59m**
Exclusive breast feeding	0.63***	2.73***	1.08	2.68***	3.89***	1.66	0.82***	0.96	0.95
Micronutrient uptake	3.17	1.49	3.47	-	1.3	1.88	1.10	1.67	0.78
Fortified food	1.18	2.01***	6.21***	-	1.67***	10.82***	1.09	0.95	0.94

*** p < 0.001;

** p < 0.05 and

*p < 0.1 & m = age of the child in months.

Furthermore, after controlling for maternal, child and environmental factors such as maternal education, age of the mothers, size of the child at birth, maternal height, and sources of drinking water as a proxy to health and sanitation of the household, the study found varying effect on the determinants on nutrition outcome of the children in terms of underweight, stunting and wasting.

For instance, in terms of the underweight, the study found that small size of the child at birth had effect to increase the likelihood of the child’s underweight condition by ages 0–5 months (OR = 3.2, p<0.001), 6–23 months old (OR = 4.29, p<0.001) and 24–59 months old (OR = 2.01, p<0.001) relative to the those who had larger weight at birth. In terms of the environmental factor, namely, source of drinking water at the household level, the study found that those households which access water from the boreholes (OR = 1.56, p<0.001) and unprotected water sources (OR = 1.69, p<0.05) were the significant predictors which increased under-weight among the children aged 6–23 months compared to their counterparts whose households were accessing piped water for domestic use. Worth noting, the women who had a height more than 1.6 m were more likely to give birth to children with low cases of underweight (OR = 0.51, p<0.001) compared to those with maternal height of less than 1.6m, significantly, among children aged 24–59 months old.

On stunting, attainment of maternal education among rural women, the study found that little education attainment at any level was found enlighten and capacitate them on significance of nutrition among their children by age categories, inadequately, more specifically those women with children aged 0–5 months and 6–23 months’ years old, as these ages were associated with a significant prevalence’s of stunting. Furthermore, considering the size of the child at birth, the study found that children who had low birth weight at birth and high prevalence of stunting between aged 0–5 months (OR = 2.58, p<0.001) and aged 6–23 months (OR = 1.77, p<0.001). However, on maternal height, it was found that mothers who had maternal height of at least 1.6m were able to give birth to children who experienced less public health challenges of stunting on ages 6–23 months (OR = 0.49, p<0.001) and 24–59 months (OR = 0.52, p< 0.1).

In terms of the wasting, it was noted that maternal education explained low likelihood of the children from the effect of significant wasting among those aged 0–5 months old (OR = 0.48, p<0.05). However, across the same age category, the study found that the children who had low weight size at birth had had a significant challenge of experiencing continued public health challenge of wasting (OR = 1.93, p<0.05) relative to the those who had a higher weight at birth. Among the children aged between 6–23 months old, the study found them suffer from wasting from despite having an average weight at birth (OR = 1.5, p<0.001) and low birth weight at birth (OR = 2.39, p<0.001). This wasting situation was similar among the children aged 23–50 months old who experienced an increased likelihood of wasting despite having an average weight at birth (OR = 1.73, p < 0.001) which was worse off among those with low birth weight (OR = 2.09, p < 0.001). Table 5 presents more details.

**Table 5:** Maternal and environment control variables estimating rural under-five children nutrition outcome by ages between 0–5, 6–23 and 24–59 months old.

Variables	Underweight			Stunting			wasting		
Maternal factors	0-5m	6-23m	24-59m	0-5m	6-23m	24-59m	0-5m	6-23m	24-59m
**Maternal education**									
No education ®	1	1	1	1	1	1	1	1	1
Primary	0.92	1.31	1.17	2.96***	-	-	0.48**	1.09	0.83
Secondary +	0.81	1.01	0.94	2.33***	-	-	0.56	0.84	1.10
**Maternal age**									
<24	1	1	1	1	1	1	1	1	1
25–34	1.23	1.15	0.92	-	-	-	1.18	0.87	0.93
35+	1.78	1.14	0.89	-	-	-	1.19	1.02	0.89
**Size of child at birth**									
Large ®	1	1	1	1	1	1	1	1	1
Average	11	1.96***	1.36**		1.49***	-	0.95	1.5***	1.73***
Small	3.2	4.29***	2.01***	2.58**	1.77***	-	1.93**	2.39***	2.09***
**Maternal height**									
<1.6m	1	1	1	1	1	1	1	1	1
> = 1.6m	0.78	0.56	0.51***	-	0.49**	0.52**	0.68	0.99	1.09
**Source of drinking water**									
Piped water®	1	1	1	1	1	1	1	1	1
Boreholes	0.76	1.56***	1.05		1.51**	-	0.97	0.98	0.77**
Open wells and streams	0.78	1.69**	0.86	0.85	-	-	1.07	0.99	0.56***

*** p < 0.001

** p < 0.05 and

*p < 0.1 & m = age of the child in months.

## Discussion

The study found that 83% of rural women indicated that they were inadequately feeding their children under five years with animal proteins rich foods, such as meat, in balanced volume to improve adequately enhance their under nutrition statuses. On the contrary, it was found that 31% of the rural women were able to give their children under five years with adequate quantities of fruits and vegetables thus on track to reducing under nutrition fight in rural Malawi.

Similarly, it is imperative to note that not only in adequate animal proteins and low uptake of fruits and vegetables do have a bearing on under-nutrition among children under five, the study also found that uptake of carbohydrates rich food is lowly consumed among the children under five with only a mean of about 27.1% of them reported by their parents that they are adequate given carbohydrate rich food stuffs. This development concurs with the recent joint UNICEF, WHO and World Bank 2017 report which reiterates that the call to eradicate under nutrition among the under-five is insufficiently pursued and this is to result in little progress in 2025 and subsequent no progress at the recount of Sustainable Development Goals in 2030 [25]. However, it is worth noting that infrequent exclusive breast-feeding practices among the under-five represented about 54% of wasting, 23.1% of under-weight and 13.3% of stunting.

In the bivariate results, the current study found that the under-nutrition condition among the children under-five in rural Malawi is significantly explained by inadequate consumption of animal proteins, fruits and vegetables, and in addition to partly and significantly by carbohydrates for wasting (WHZ <-2) and stunting (HAZ<-2). This finding concurs with a study that postulated that inadequate consumption of fruits and vegetables, more especially among the children, to have unhealthy and unbalanced dietary food consumption which subsequently increased vulnerability to various diseases and mortality experiences in worst case scenario [26].

On the same note, it was also noted that infrequent practice among women in administering exclusive breast feeding was associated with a significant relation towards underweight and stunting exclusive among rural children under five children in Malawi. This implies that little variations do exist on the impact of different dietary consumption practices on the quality of nutrition status assumed by the children. A study on social determinants and under nutrition among the under-five argues that minimising suboptimal feeding social care such as breastfeeding has a direct impact to improve the quality of nutritional status of the children, hence, reduce their under nutrition condition completely [27].

In a Multivariate analysis, the nested logistic regression found that exclusive breast feeding remained consistent and significant predictors of reducing wasting, underweight and stunting among children in rural Malawi after controlling for parental socio-economic status. A similar study in Vietnam found that delay in initiation of the breast feeding among the under-five children is a major catalyst that increased the levels of under nutrition [28]. This implies that deliberate and increased intensity administration of exclusive breast feeding has an impetus of reducing under nutrition among children. As such, controlling for socio-economic and environmental factors affecting women adherence to have a consistent behavioural to practice exclusive breast feeding, is fundamental to deal with persistent under nutrition affecting children [29].

Contrary to this, it was found that inadequate consumption of carbohydrates and micronutrients supplement were the most significant predictors of underweight and stunting among the children under five in rural Malawi. The result is in support of an earlier assertion that poor feeding practices with no balanced diet has a direct effect of increasing under nutrition [30,31].

On the same note, in general among all children under five years, it was also found that fruits and vegetables inadequate consumption among the children increased their wasting and underweight under-nutrition status. In this aspect, it was observed that if children are not repeatedly exposure to fruits and vegetables during their first years of life, their consumption preference of fruits and vegetables become lower than necessary [32]. This consequently affects their quality of assumed health during their early days of life [33].

In the adjusted models, the study found that despite attainment of the maternal primary school education, their children still experience significant and increased levels of underweight and stunting. However, among the men, despite paternal education was found to be associated with a significant reduction in wasting under-nutrition status among children, it was found to have a contrary impact in increasing underweight under nutrition status among the rural children.

In the same way, the study found that despite women reported that they had a postnatal care services access after childbirth, it was found that their visits were relatively less in reducing underweight and stunting of their children under five respectively. It is earlier argued that not only improved socio-economic factors do provide an opportunity cost for communities to purchase quality of health care but also availability and health facility capacity with the capacity of meeting the emergent needs of the people, such as nutritional information [34,35] and long–run improved quality of life among the vulnerable children. Additionally, the study found that the higher the parity the women acquire the likelihood of their children in suffering an increased level of underweight and stunting increases significantly. It is argued that as in appropriate feeding practices that women subject their children on as a result of increased burden of children affects under nutrition status [3,36].

Therefore, based on the decomposed estimates of the nutrition outcome, it has been noted that the lower the size of the child at births, the more it contributes towards the worst nutrition outcome across the ages 0–5, 6–23 and 24–59 months. Such findings concur with Nabwera and others who noted that low birth weight among the children have a bearing on their growth and persistent underweight, stunting and wasting experiences [37]. Furthermore, the study found that access to water from the boreholes and open wells or streams by the households, remained the consistent predictors affecting underweight and stunting among children aged 6 months or higher. This implies that water quality that the households are accessing in the country, still contributes towards worst nutrition status of the children. It is because of this continued public health challenges that the need to ascertain improved quality water and supply in addition to sanitation and hygiene practices at the household levels, more especially in developing countries, is fundamental to improve nutrition statuses of the children during the first 1000 days of life [38, 39].

## Conclusion

Exclusive breast feeding behaviour among women on their children under-five years after controlling for the parental socio-economic statuses is found to be the major consistent and significant predictor influencing cases of children under five years suffer from under nutrition public health challenges in rural Malawi. Furthermore, the current study found that despite mothers reported adequacy in feeding the children under five in fruits and vegetables, carbohydrates composed meals and micronutrient supplements, such dietary consumption still have little effect to reduce cases of wasting, underweight and stunting among children in rural Malawi, more especially during first 59 months of live. It is important to note that the need to implement extensive pro-rural under-five nutritional and health education advocacy training on dietary diversity and food value compositions that would benefit the children under five, if and only if the persistent public health challenges that emanates as a consequence of under nutrition is to be comprehensively eradicated among the rural children under five in Malawi. For instance, the inconsistent dietary practices such as exclusive breast feeding among the children under five aged 0–5 months, not only still have slow progress in reducing underweight and wasting, but also little impact to significantly reduces cases of underweight and stunting among their counterparts aged 6–23 months old. Furthermore, use of fortified feds among the rural women as food supplements for their children was associated with little impact to reduce cases of both underweights and stunting significantly among children aged 6–23 and 24–59 months old. Therefore, the need to enhance knowledge on importance of dietary practices among rural women is key if rural children public health challenges related to nutrition is to be served in the first 59 months of life and gives them an impetus to develop and assist greatly towards socio-economic progress of Malawi.
